# Listeriosis Cases and Genetic Diversity of Their *L. monocytogenes* Isolates in China, 2008–2019

**DOI:** 10.3389/fcimb.2021.608352

**Published:** 2021-02-19

**Authors:** Binghuai Lu, Junwen Yang, Chunyan Gao, Dong Li, Yanchao Cui, Lei Huang, Xingchun Chen, Duochun Wang, Aiping Wang, Yulei Liu, Yi Li, Zhijun Zhang, Mingyuan Jiao, Heping Xu, Yu Song, Baoqing Fu, Lili Xu, Qing Yang, Yongzhong Ning, Lijun Wang, Chunmei Bao, Guolan Luo, Hua Wu, Tongshu Yang, Chen Li, Manjuan Tang, Junrui Wang, Wenchen Guo, Ji Zeng, Wen Zhong

**Affiliations:** ^1^Laboratory of Clinical Microbiology and Infectious Diseases, Department of Pulmonary and Critical Care Medicine, China-Japan Friendship Hospital, Beijing, China; ^2^Center for Respiratory Diseases, China-Japan Friendship Hospital, Beijing, China; ^3^Laboratory of Clinical Microbiology and Infectious Diseases, National Clinical Research Center of Respiratory Diseases, Beijing, China; ^4^Department of Laboratory Medicine, Zhengzhou Key Laboratory of Children’s Infection and Immunity, Children’s Hospital Affiliated to Zhengzhou University, Zhengzhou, China; ^5^Department of Laboratory Medicine, Tangshan Maternal and Child Health Care Hospital, Tangshan, China; ^6^Department of Laboratory Medicine, Civil Aviation General Hospital, Beijing, China; ^7^Department of Laboratory Medicine, Peking University First Hospital, Beijing, China; ^8^Department of Laboratory Medicine, People’s Hospital of Guangxi Zhuang Autonomous Region, Nanning, China; ^9^National Institute for Communicable Disease Control and Prevention, State Key Laboratory for Infectious Disease Prevention and Control, Chinese Centre for Disease Control and Prevention, Beijing, China; ^10^Department of Laboratory Medicine, Beijing Anzhen Hospital, Beijing, China; ^11^Department of Laboratory Medicine, Henan Provincial People’s Hospital, Zhengzhou, China; ^12^Department of Laboratory Medicine, Tai’an City Central Hospital, Tai'an, China; ^13^Department of Laboratory Medicine, Beijing Tongzhou District Maternal and Child Healthcare Hospital, Beijing, China; ^14^Department of Laboratory Medicine, First Affiliated Hospital of Xiamen University, Xiamen, China; ^15^Department of Laboratory Medicine, Daqing Oilfield General Hospital, Daqing, China; ^16^Department of Laboratory Medicine, Fifth People’s Hospital of Chengdu, Chengdu, China; ^17^Department of Laboratory Medicine, First Affiliated Hospital, College of Medicine, Zhejiang University, Hangzhou, China; ^18^Department of Laboratory Medicine, Chui Yang Liu Hospital Affiliated to Tsinghua University, Beijing, China; ^19^Department of Laboratory Medicine, Beijing Tsinghua Chang Gung Hospital, Tsinghua University, Beijing, China; ^20^Clinical Laboratory Medical Center, The Fifth Medical Center of Chinese PLA General Hospital, Beijing, China; ^21^Department of Laboratory Medicine, Fourth Affiliated Hospital of Guangxi Medical University, Liuzhou, China; ^22^Department of Laboratory Medicine, Hainan General Hospital, Haikou, China; ^23^Department of Laboratory Medicine, The Affiliated Tumor Hospital of Harrbin Medical University, Harbin, China; ^24^Department of Laboratory Medicine, Liuyang City Traditional Chinese Medicine Hospital, Liuyang, China; ^25^Department of Laboratory Medicine, Xiangtan Central Hospital, Xiangtan, China; ^26^Department of Laboratory Medicine, Affiliated Hospital of Inner Mongolia Medical University, Hohhot, China; ^27^Department of Laboratory Medicine, Weifang People’s Hospital, Weifang, China; ^28^Department of Laboratory Medicine, Wuhan Pu Ai Hospital of Huazhong University of Science and Technology, Wuhan, China; ^29^Department of Laboratory Medicine, Ningde Hospital, Fujian Medical University, Ningde, China

**Keywords:** neonatal listeriosis, multilocus sequence typing, antibiotic resistance profile, isteriosis, *Listeria monocytogenes*

## Abstract

Listeriosis, caused by *Listeria monocytogenes*, is a severe food-borne infection. The nationwide surveillance in China concerning listeriosis is urgently needed. In the present study, 144 *L. monocytogenes* isolates were collected from the samples of blood, cerebrospinal fluid (CSF), and fetal membrane/placenta in China for 12 years from 2008 to 2019. We summarized these listeriosis patients’ demographical and clinical features and outcomes. The susceptibility profile for 12 antibiotics was also determined by the broth microdilution method. Multilocus sequence typing (MLST) and serogroups of these listeria isolates were analyzed to designate epidemiological types. We enrolled 144 cases from 29 healthcare centers, including 96 maternal-neonatal infections, 33 cases of bacteremia, 13 cases of neurolisteriosis, and two cutaneous listeriosis. There were 31 (59.6%) fetal loss in 52 pregnant women and four (9.8%) neonatal death in 41 newborns. Among the 48 nonmaternal-neonatal cases, 12.5% (6/48) died, 41.7% (20/48) were female, and 64.6% (31/48) occurred in those with significant comorbidities. By MLST, the strains were distinguished into 23 individual sequence types (STs). The most prevalent ST was ST87 (49 isolates, 34.0%), followed by ST1 (18, 12.5%), ST8 (10, 6.9%), ST619 (9, 6.3%), ST7 (7, 4.9%) and ST3 (7, 4.9%). Furthermore, all *L. monocytogenes* isolates were uniformly susceptible to penicillin, ampicillin, and meropenem. In summary, our study highlights a high genotypic diversity of *L. monocytogenes* strains causing clinical listeriosis in China. Furthermore, a high prevalence of ST87 and ST1 in the listeriosis should be noted.

## Introduction

Listeriosis is a severe foodborne bacterial infection, caused by *Listeria monocytogenes*, an intracellular Gram-positive facultative bacillus (Vázquez-Boland et al., 2001; Moura et al., 2016; Charlier et al., 2017).

Several sub-typing methods have been developed for understanding the microbiological characterization of *L. monocytogenes* isolates. PCR serogrouping is often performed in the epidemiological investigation (Doumith et al., 2004; Leclercq et al., 2011). Namely, this PCR serogrouping method targets the DNA fragments *ORF2110*, *ORF2819*, *lmo1118*, and *lmo0737*, and classifies Listeria species into IIa, IIb, IIc, IVb, and L (Leclercq et al., 2011). Furthermore, multilocus sequence typing (MLST), based on seven housekeeping genes, is also a powerful and extensively-used epidemiological typing tool and allows for the classification of most clinical *L. monocytogenes* isolates into multiple clonal complexes (CCs) (Moura et al., 2016; Wu et al., 2016). In addition, *L. monocytogenes* can be classified into four distinct evolutionary lineages (denoted I to IV) with most isolates grouping into lineages I and II (Cardenas-Alvarez et al., 2019; Fan et al., 2019).

Nowadays there is no specific reference diagnosis and treatment recommended for listeriosis in China (Li et al., 2018). The first-line treatment strategy for invasive listeriosis is based on a synergistic association of amoxicillin (or aminopenicillin) plus gentamicin (Hof, 2004; Fernández Guerrero et al., 2012). Furthermore, in case of the allergy to beta-lactams, trimethoprim-sulfamethoxazole (TMP-SMZ), rifampin, and carbapenems have been proposed as potential alternatives (Morvan et al., 2010; Fernández Guerrero et al., 2012; Dickstein et al., 2019). The antibiotic susceptibility profiles of *L. monocytogenes* strains might differ geographically and over time (Chenal-Francisque et al., 2014; Luque-Sastre et al., 2018; Yan et al., 2019; Zhang et al., 2019). Continuous monitoring of the epidemiological changes is crucial for the prevention and treatment of listeriosis.

To date, rare original studies have been conducted in mainland China to investigate the epidemiology of *L. monocytogenes* (Feng et al., 2013; Wang et al., 2013; Wang et al., 2015; Fan et al., 2019; Lu et al., 2019; Zhang et al., 2019; Chen et al., 2020). The national foodborne disease surveillance system was set-up in 2011, and starting from 2013, the surveillance of listeriosis sporadic cases was included in the national surveillance plan only in limited provinces (Pei et al., 2015; Li et al., 2018). A multi-center, longitudinal, and national epidemiological study on listeriosis is lacking and the recent trend of listeriosis remains unclear. Herein, we investigated the clinical features, molecular diversity and antimicrobial resistance profiles of 144 *L. monocytogenes* isolates over 12 years in mainland China. Our results are also compared to globally-reported data.

## Methods

### Ethical Statement

The institutional review boards at the Civil Aviation General Hospital approved the study protocol. For the retrospective study, the review board waived the need for informed consent.

### Case Definition

We classified the listeriosis as maternal-neonatal listeriosis (including maternal infection, neonatal infection, and both), nonmaternal-neonatal listeriosis (including bacteremia, neurolisteriosis, or other forms) as defined previously (Goulet et al., 2012; Charlier et al., 2017). Maternal-neonatal listeriosis was defined when *L. monocytogenes* was isolated in pregnant women, fetuses, or infants during the first 28 days of life (Goulet et al., 2012; Li et al., 2018). When *L. monocytogene*s was isolated from samples of both the mother and her newborns and/or fetuses, the event was only counted as a single case (Fan et al., 2019). Nonmaternal-neonatal listeriosis patients were defined as those who were not pregnant women or newborns. Bacteremia was defined when *L. monocytogenes* was isolated from blood culture, without neurolisteriosis or maternal-neonatal infection. Neurolisteriosis was defined when *L. monocytogenes* was isolated from the cerebrospinal fluid (CSF) (Charlier et al., 2017). When the outcome was death for a nonmaternal-neonatal patient or abortion/miscarriage or newborn death for a maternal-neonatal patient, this was counted as an adverse outcome.

Furthermore, in accordance with the previous reports, the risk factors of listeriosis are defined as follows: cancer (non-hematological malignancy and hematological malignancy), diabetes, HIV/AIDS, solid organ transplantation, dialysis, liver diseases and cirrhosis, valvulopathy (living with abnormal heart valves or a prosthetic valve), inflammatory diseases (ulcerative colitis, systemic lupus erythematosus (SLE) and Crohn’s and rheumatoid arthritis), age≧65 years and pregnancy (Goulet et al., 2012; Morgand et al., 2018).

### Bacterial Strains Collection

**Figure 1:**
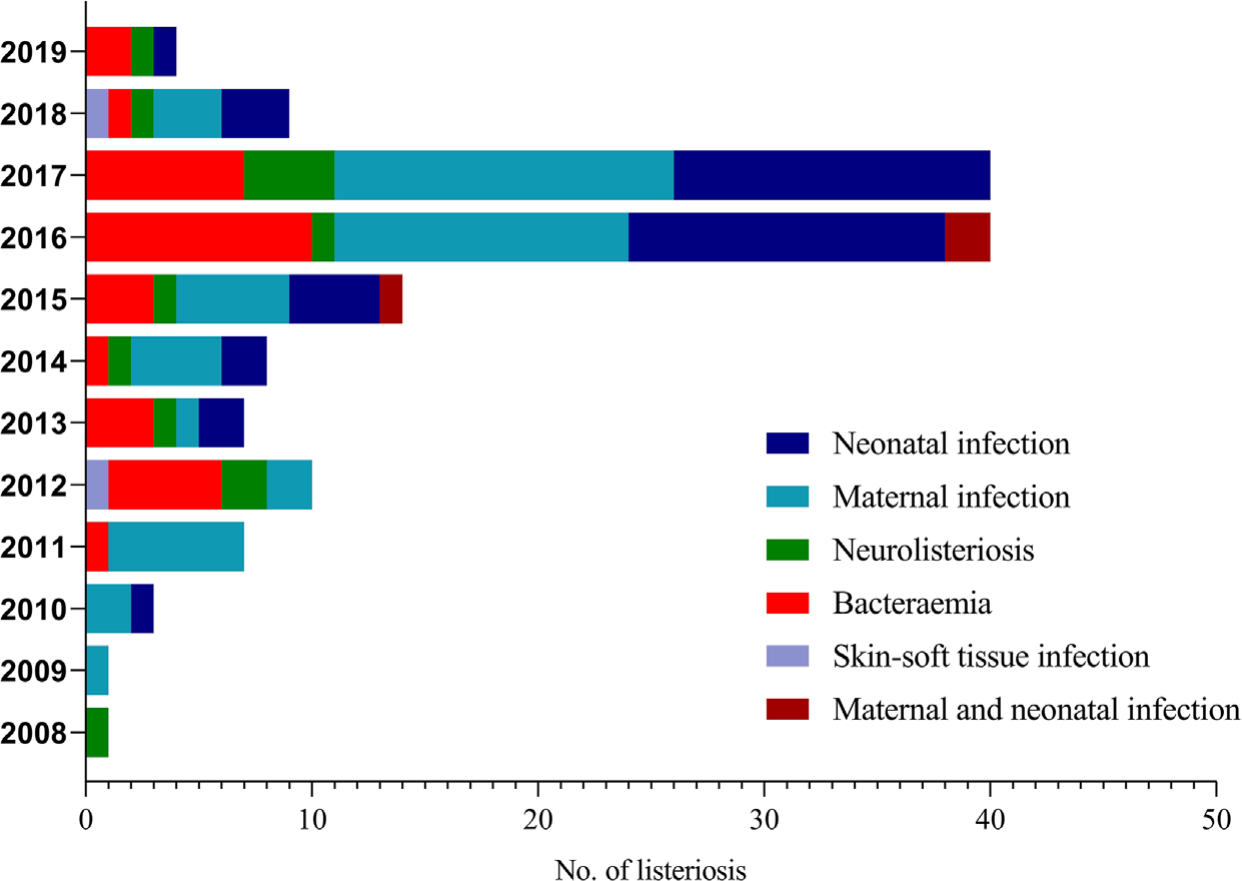
Distribution of infection types of 144 listeriosis cases in China between 2008 and 2019.

During a 12-year period from 2008 to 2019, 144 *L. monocytogenes* isolates in this study were recovered from infected neonates, mothers and non-pregnant population who visited the following medical institutes, with cities/provinces indicated in parentheses: Beijing Anzhen Hospital (Beijing), Beijing Tongren Hospital (Beijing), Beijing Tsinghua Changgung Hospital (Beijing), Beijing Tongzhou District maternal and child healthcare hospital (Beijing), Civil Aviation General Hospital (CAGH, Beijing), Fifth Clinical Center of PLA General Hospital (Beijing), Peking University First Hospital (Beijing), Peking University Third Hospital (Beijing), Ningde Hospital, Fujian Medical University (Fujian), First Affiliated Hospital of Xiamen University (Fujian), Xiamen Maternal and Child Health Hospital (Fujian), Ganshu Dingxi People’s Hospital (Ganshu), Fourth Affiliated Hospital of Guangxi Medical University (Guangxi), People’s Hospital of Guangxi Zhuang Autonomous Region (Guangxi), Hainan General Hospital (Hainan), Qinhuangdao Maternal and Child Health Care Hospital (Hebei), Tangshan Maternal and Child Health Care Hospital (Hebei), Daqing Oilfield General Hospital (Heilongjiang), Harbin Cancer Hospital (Heilongjiang), Henan Provincial People’s Hospital (Henan), Children’s Hospital Affiliated to Zhengzhou University (Henan), Wuhan Pu Ai Hospital of Huazhong University of Science and Technology (Hubei), Liuyang city traditional Chinese medicine hospital (Hunan), Xiangtan Central Hospital (Hunan), Affiliated hospital of Xuzhou medical university (Jiangshu), Affiliated hospital of Inner Mongolia medical university (Neimenggu), Weifang People’s Hospital (Shandong), Fifth People’s Hospital of Chengdu (Sichuan), and First Affiliated Hospital, College of Medicine, Zhejiang University (Zhejiang). The detailed geographical locations of *L. monocytogenes* isolates from listeriosis patients are shown in **Figure S1**. Our *L. monocytogenes* isolates were recovered from infected neonates, mothers, and non-pregnant populations. Besides, when *L. monocytogenes* strains from different anatomic sites (CSF, blood, etc.), or concurrently isolated from both mother and newborn, had identical morphological characteristics and identification results, only one strain was chosen for further study. Therefore, a total of 144 non-redundant *L. monocytogenes* isolates were enrolled in the current study. The demographic characteristics, the geographical distribution of the listeriosis patients, and detailed information on these strains are shown in **Table 1** and **Figure 1** and **Figure S1**.

**Table 1:** Clinical and molecular features of 144 *Listeria monocytogenes* isolates in China.

Infection type	Total number	n%	Underlying diseases	Gender	Adverse outcome(No, %)	STs	Serogroups
**Maternal-neonatal infection**	96	66.7%					
Maternal infection	52	36.1%		female (52)	stillbirth/abortion (31, 59.6%)	ST1(5); ST3(5); ST5(2); ST7(2); ST8(3); ST14(2); ST87(14); ST(3); ST101(2); ST121(2); ST145; ST155(2); ST224(2); ST330; ST619(5); ST1225	IIa(16); IIb(30); IVb(6)
Neonatal infection	41	28.5%		male (21); female (20)	neonatal death (4, 4.2%)	ST1(3); ST2; ST3; ST5; ST7(3); ST8(3); ST14; ST87(18); ST91; ST101(2); ST155; ST224(2); ST298; ST619(2); ST621	IIa(5); IIb(24); IVb(12)
Maternal and neonatal infection	3	2.1%		newborns (male, 1; female, 2)	No	ST1; ST87(2)	IIb(2); IVb
**Non maternal-neonatal infection**	48	33.3%					
Bacteremia	33	22.9%	≧65 (12); malignancy (10); diabetes (7); autoimmune disorders/hormone user (6); cirrhosis; CKD.	male (21); female (12)	died (4, 12.1%)	ST1(6); ST2; ST3; ST4; ST5(2); ST7(2) ST8(3); ST9; ST87(9); ST101; ST121; ST145; ST177; ST307; ST619(2)	IIa(8); IIb(15); IIc; IVb(9)
Neurolisteriosis	13	9.0%	≧65 (3); autoimmune disorders/hormone user (SLE, 2); CKD (2); leukemia.	male (6); female (7)	died (2, 15.4%)	ST1(3); ST8; ST87(4); ST91; ST101; ST155; ST298; ST621	IIa(5); IIb(4); IVb(4)
Skin-soft tissue infection	2	1.4%	diabetes	male; female	No	ST87 (2)	IIb (2).

Malignancy including Hodgkin’s lymphoma, myeloma, acute B lymphocytic leukemia, acute monocytic leukemia, chronic myeloid leukemia, breast, lung, liver and prostate cancers, and multiple metastases. Autoimmune disorders/hormone user including ulcerative colitis, connective tissue disease, systemic lupus erythematosus (SLE) and lupus nephritis, thrombocytopenia (idiopathic thrombocytopenic purpura (ITP), and stem cell transplantation. CKD, chronic kidney diseases; ST, sequence types.

### *L. monocytogenes* Identification by Using Matrix-Assisted Laser Desorption Ionization-Time of Flight Mass Spectrometry (MALDI-TOF MS)

Following the protocol described in our previous study (Li et al., 2020), *L. monocytogenes* isolates were inoculated onto sheep blood agar plates (BAPs) and incubated overnight at 35°C with 5% CO_2_. Afterward, the colonies were collected and identified to the species level based on colony morphology and MALDI-TOF MS (Bruker Daltonik, Bremen, Germany) in line with the recommended protocol, and 16S rRNA sequencing if necessary.

For MALDI-TOF MS methods, the isolates were primarily identified using a direct transfer method. Briefly, the fresh colonies on the BAPs were picked up with an inoculation loop, smeared on an MTP 384 steel target plate, coated with a matrix solution of a-cyano-4-hydroxycinnamic acid (HCCA) in 50% acetonitrile with 2.5% trifluoroacetic acid (TFA). If no reliable result was obtained, an ethanol/formic acid extraction method was then applied. One loop of bacterial mass was suspended in deionized water (300 µl), and pure ethanol (900 µl) was added. The suspension was mixed for 1 min using a vortex mixer. The cell suspension was centrifuged (13,000 rpm for 2 min). The supernatant was discarded. Then, the pellet was dried and resuspended with 70% formic acid (50 µl) with thorough mixing, and 50-µl acetonitrile was added. After centrifugation (13,000 rpm for 2 min), 1-µl pellet was applied on a steel plate, dried at room temperature, and coated with 1-µl HCCA. The identification was matched with the Bruker spectra library program (version 4.0.0.1, 5,627 entries), preinstalled in the Bruker Biotyper device (version 3.1; Bruker.1). The identification score criteria were used: scores ≧2.0 were considered to be reliable to the species level; scores ≧1.7 and <2.0 were considered reliable to the genus level, and scores <1.7 was interpreted as no identification.

### PCR Template Preparation

Genomic DNA was extracted from each Listeria strain using the genomic DNA purification kit (Tiangen Biotech, Beijing, China) following the manufacturer’s instructions. Briefly, protoplasts were prepared by incubating the fresh strains in a microcentrifuge tube with 1-ml saline; the solution was prepared to a concentration of 1 McFarland, and centrifuged at 12,000 rpm for 1 min. The supernatant was discarded. 600-µl PBS buffer and 6-µl (10 U/µl) cell wall breaking enzyme (Tiangen biochemical technology co., Ltd., China) were added into a microcentrifuge tube, and thoroughly mixed and incubated at 37°C for 120 min. After vortexing, 400 µl of 2-µm acid-washed glass beads were added and further vortexed. Extracted DNAs were dissolved in TE buffer and stored at -20°C until used as PCR templates.

### 16S rRNA Sequencing

Identification of Listeria species through the amplification of the specific 16S rRNA region was performed using two universal primers: 27f primer formulations (5’-AGAGTTTGATCCTGGCTCAG-3’) and1492r primer (5’-TACCTTGTTACGACTT-3’). The PCR products were purified and sequencing was performed by using ABI 3730 DNA analyzer. The sequences were compared to the database in NCBI Genbank (http://www.ncbi.nlm.nih.gov) using the BLAST algorithm.

### PCR Serogrouping

All the *L. monocytogenes* strains were serogrouped by amplifying the following target genes: *prs*, *lmo0737*, *lmo1118*, *ORF2110*, and *ORF2819*, using primer pairs and amplification conditions described previously (Doumith et al., 2004).

### MLST, Phylogenetic, and Epidemiological Analyses

MLST was performed by amplifying and sequencing the internal fragments of the following seven housekeeping genes in line with the MLST sequence type database http://bigsdb.web.pasteur.fr/listeria/ (Moura et al., 2016) and Ragon et al.’s scheme (Ragon et al., 2008): *abcZ* (ABC transporter), *bglA* (beta-glucosidase), *cat* (catalase), *dapE* (succinyl diaminopimelate desuccinylase), *dat* (D-amino acid aminotransferase), *ldh* (L-lactate dehydrogenase), and *lhkA* (histidine kinase). The following cycling and conditions were applied for amplifying the above genes: 94°C for 4 min followed by 35 cycles of 94°C for 30 s, 52°C for all genes except for *bglA* (45°C) for 30 s, and 72°C for 2 min. Amplifications were finalized with a 10-min 72°C step.

The PCR products were sequenced in both directions (TaKaRa, Dalian, China). Contiguous nucleotide sequences were assembled with MEGA software (Version 10.0.0.5), and sequence variants were designated allele profiles. Isolates with identical allelic profiles were assigned to the same sequence type (ST). The STs, allelic numbers, and CCs were subsequently further determined by querying the above database. A minimum-spanning tree was constructed to illustrate the genetic relationships between STs and CCs using the allelic differences between isolates of the seven housekeeping genes and BioNumerics software (version 7.5, Applied Math, Belgium).

### Antibiotic Susceptibility Testing (AST)

The broth microdilution method was used to determine the minimal inhibitory concentrations (MICs) of all strains to penicillin, ampicillin, TMP-SMX, erythromycin, clindamycin, meropenem, moxifloxacin, ciprofloxacin, rifampin, gentamicin, tetracycline, and vancomycin. Cation-adjusted Mueller-Hinton broth, supplemented with lysed horse blood (5% v/v), was provided by the Tianjin Jinzhang Science and Technology Development, and the above antibiotics were manufactured by Dalian Meilun Biotechnology Co., Ltd. The AST was conducted under 35°C for 20–24 h in ambient air following the incubation conditions and breakpoints set by the Clinical and Laboratory Standards Institute (CLSI) (CLSI, 2015). *S. pneumoniae* ATCC 49619 was used for quality control.

### Statistical Analysis

Statistical analyses were conducted using GraphPad Prism version 8.0.1. All antibiotic susceptibility data were analyzed using WHONET 5.6 software, and MIC50 and MIC90 were also calculated.

## Results

### *L. monocytogenes* Strains

**Figure 2:**
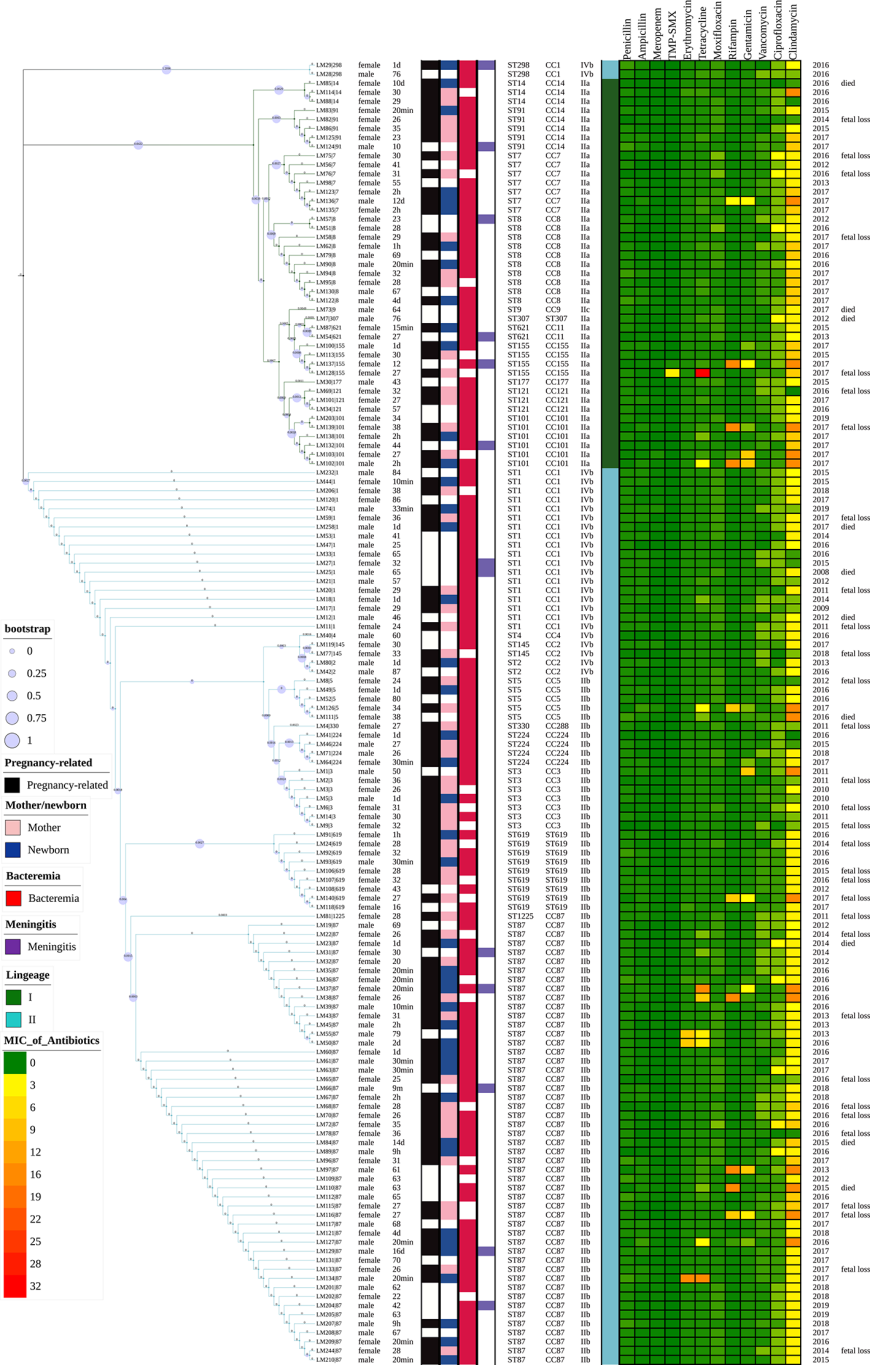
Representation of analysis and phylogeny of *L. monocytogenes* strains collected from patients with listeriosis in 15 cities/provinces in China. A complete picture of the phylogeny was constructed by the maximum likelihood method using seven housekeeping genes of multilocus sequence typing (MLST) of the above strains using MEGA (Version 10.0.5) and iTOL v4. from left to right: 1 Sex; 2 Age; 3 Pregnancy or not; 4 Mother/newborn (both mothers and newborns were infected in the cases of LM44, 39, 35); 5 Bacteremia; 6 Meningitis; 7 Sequence types; 8 Clonal complexes; 9 Serogroups; 10 Lineage; 11–22 MICs of 12 antibiotics (µg/ml); 23 Year of isolation of *L. monocytogenes* strains; 24 Adverse outcomes.

We enrolled 144 listeriosis cases from 29 healthcare centers, including 96 maternal-neonatal infections and 48 nonmaternal-neonatal infections (including 33 cases of bacteremia, 13 cases of neurolisteriosis, and two cases of skin-soft infection) (**Figure 1** and **Table 1**). Of them, 108 (75.0%) and 21 (14.6%) were positive in blood and CSF culture, respectively, and there were eight cases (three adults and five newborns) in whom *L. monocytogenes* were isolated from both bloodstream and CSF, seen in **Table 1** and **Figure 2**. The characteristics of two cutaneous cases were detailed in **Table 2**. All cases occurred sporadically and we did not find the hospital source in these listeriosis patients. All these patients were hospitalized during the diagnosis and treatment.

**Table 2:** Summary of the two cutaneous cases of listeriosis.

Patients No.	LM109	LM202
Gender	male	female
Specimen	Incision exudate	Pus
Age	63	22
Genoserotype	IIb	IIb
Sequence typing	87	87
Clonal complexes	CC87	CC87
Linage	I	I
Pregnancy	No	No
Underlying diseases	Diabetes	No
Outcomes	Recovery	Recovery
Year of onset	2012	2018
Outcomes	Recovery	Recovery
Blood culture	negative	negative
**Antibiotic resistance testing**		
Erythromycin (µg/ml)	0.25	0.25
Clindamycin (µg/ml)	4	4
Tetracycline (µg/ml)	0.5	0.25
Penicillin (µg/ml)	0.5	0.25
Ampicillin (µg/ml)	0.25	0.125
Meropenem (µg/ml)	0.125	0.125
TMP-SMX (µg/ml)	0.032/0.6	0.032/0.6
Moxifloxacin (µg/ml)	0.25	0.5
Ciprofloxacin (µg/ml)	0.5	1
Rifampin (µg/ml)	0.125	0.125
Gentamicin (µg/ml)	0.25	0.25

**Figure 3:**
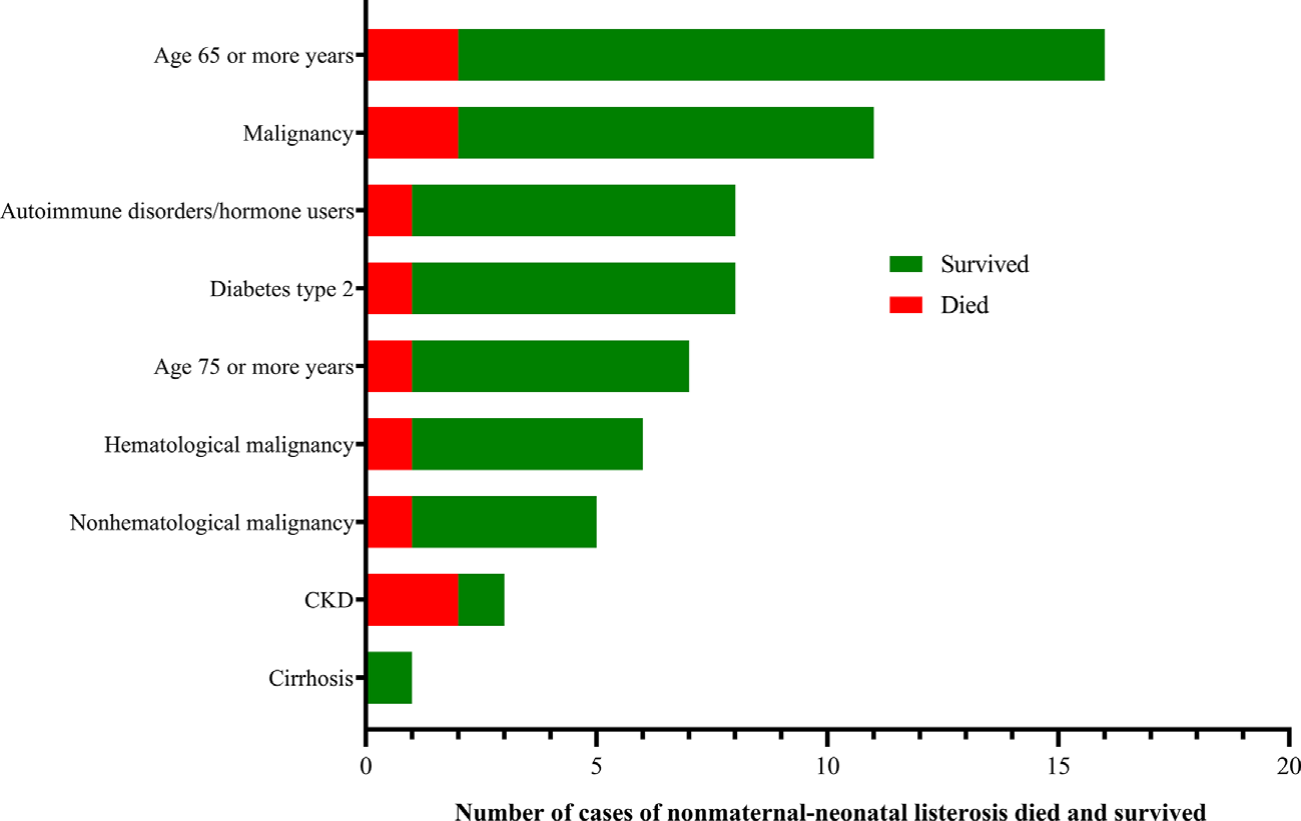
Risk factors of 48 nonmaternal-neonatal listeriosis cases.

Forty-one (41/144, 28.5%) adverse outcomes were reported. Among 52 maternal listeriosis cases, 31 (59.6%) experienced abortion or miscarriage. For 48 non-maternal-neonatal cases, the median age was 51.7 years (range, 9m-87 years), and 41.7% (20/48) were female. Patients older than 65 years accounted for 31.3% (15/48). There were 64.6% (31/48) listeriosis cases occurred in patients having the risk factors for this foodborne disease. As shown in **Table 1** and **Figure 3**, 10 (10/48, 20.8%) patients had concurrent neoplasms: leukemia, multiple myeloma, liver cancer, breast cancer, and abdominal malignant metastases;10 with autoimmune diseases: SLE (4), ulcerative colitis (1), connective tissue disease, thrombocytopenia; other comorbidities included diabetes mellitus, cirrhosis, and chronic kidney diseases (CKDs). The mortality rates in nonmaternal-neonatal cases were similar for bacteremia (12.1%, 4/33) and neurolisteriosis (15.4, 2/13), respectively, as shown in **Table 1**. No long-term follow-up was available.

### PCR Serogrouping

PCR serogrouping divided the 144 clinical isolates into four serogroups. The most prevalent serogroup was serogroup IIb with a frequency of 52.7% (76 strains), followed by IIa (42, 29.2%), and IVb (25, 17.4%), respectively. Serogroup IIc was represented only by one isolate.

### Genetic Diversity as Shown by MLST Analysis

**Figure 4:**
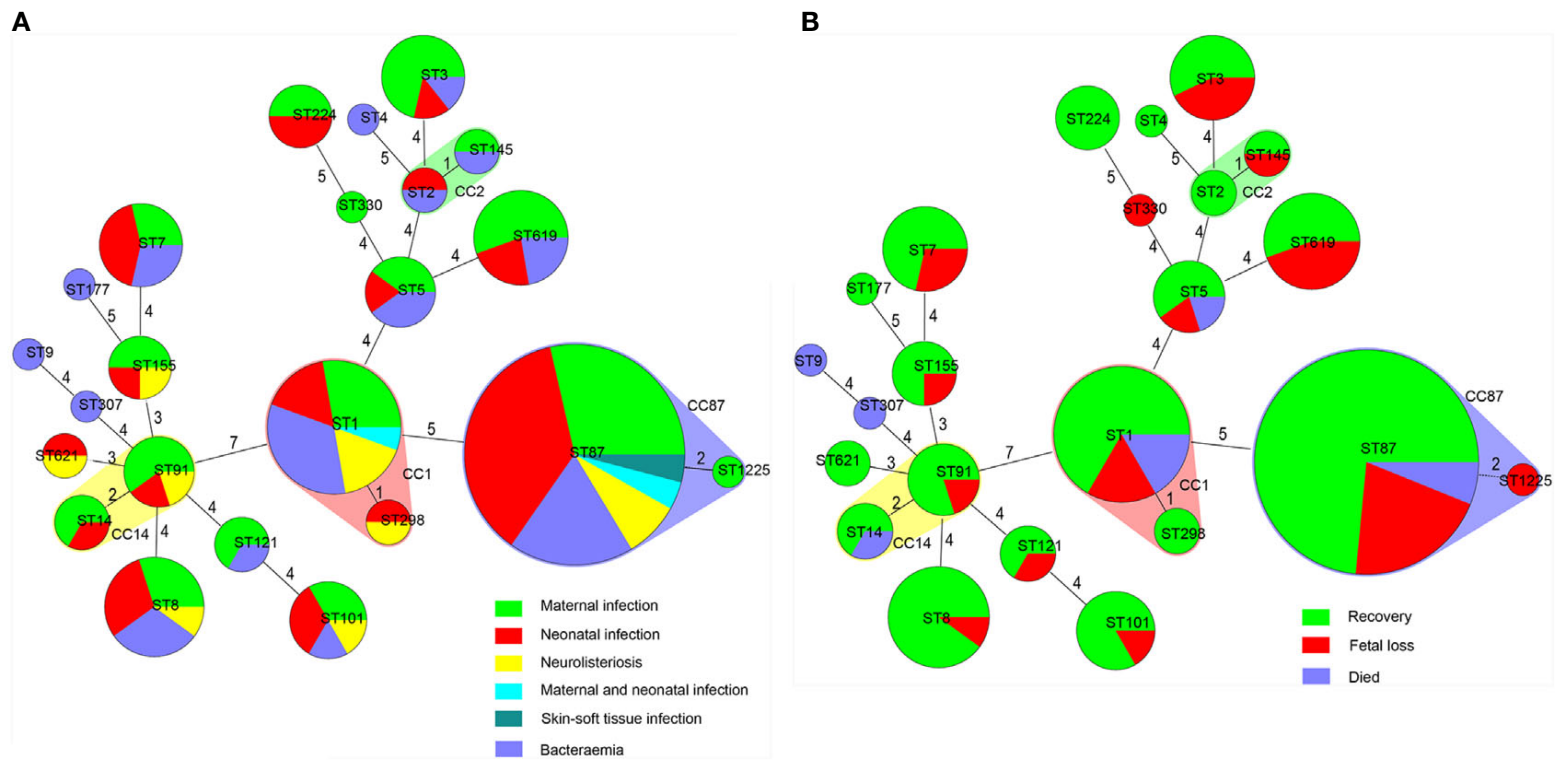
Correlation between sequence type (ST) of *L. monocytogenes* strains, types as well as outcomes of patients with listeriosis. Minimum spanning tree analysis of *L. monocytogenes* strains conducted according to ST, demonstrating the relationships among 144 strains collected from patients with listeriosis in 15 cities/provinces. in the minimum spanning tree, the STs are displayed as circles; the size of each circle indicates the number of strains within this particular type. The founder ST was defined as that with the highest number of single-locus variants. STs varying by two alleles in their multilocus sequence typing (MLST) profiles (single-locus variants) are arranged in circles around the primary founder ST. **(A)** Relationship between infection types of the patients and STs of all 144 *L. monocytogenes* strains. **(B)** Relationship between outcomes of the patients and STs of all 144 *L. monocytogenes* strains.

By MLST, 23 sequence types (STs) were distinguished, 101 and 43 of which belong to lineage I and lineage II, respectively. As demonstrated in **Figures 2** and **4**. The most prevalent STs were ST87 (49 strains, 34.0%), ST1 (18, 12.5%), ST8 (10, 6.9%), ST619 (9, 6.3%), ST7 (7, 4.9%), and ST3 (7, 4.9%). Furthermore, 69.4% (100/144) of all strains were represented by these six STs (ST87, 1, 8, 619, 7, and 3) that are widely distributed, and six STs (ST 4, 9, 177, 307, 330, 1225) were represented only by one strain.

### Antibiotic Susceptibility Testing

**Figure 5:**
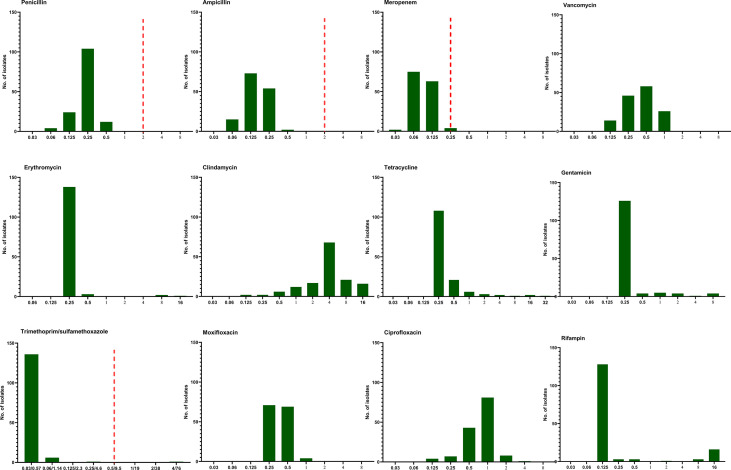
The distribution of the Minimum inhibitory concentration (MIC) 144 clinical unique strains of *L. monocytogenes* for 12 antibiotics. Redline: the breakpoint for *L. monocytogenes* by CLSI.

The antimicrobial susceptibility and MIC results for 12 antimicrobial agents against all 144 *L. monocytogenes* strains are presented in **Table 3** and **Figure 5**. Our results demonstrate that, in line with the breakpoint established by CLSI, all *L. monocytogenes* strains were uniformly susceptible to penicillin, ampicillin, and meropenem, and one strain were nonsusceptible to TMP-SXT (MIC=4/76µg/ml).

**Table 3:** Antimicrobial susceptibility and minimum inhibitory concentrations of 144 isolates of *L. monocytogenes*.

Antimicrobials	Breakpoint by CLSI	Susceptibility		MIC		
		S (%)	NS(%)	MIC50 (µg/ml)	MIC90 (µg/ml)	Range (µg/ml)
Penicillin	≦2	144 (100)	0	0.25	0.5	0.06-0.5
Ampicillin	≦2	144 (100)	0	0.125	0.25	0.06-0.5
TMP-SMX	≦0.5/9.5	143 (99.3)	0.7	≤0.03/0.57	≤0.03/0.57	≤0.03/0.57~4/76
Meropenem	≦0.25	144 (100)	0	0.06	0.125	0.03~0.25
Erythromycin	ND	ND	ND	0.25	0.25	0.25~16
Vancomycin	ND	ND	ND	0.5	1	0.125~1
Tetracycline	ND	ND	ND	0.25	0.5	0.25~32
Clindamycin	ND	ND	ND	4	16	0.125~16
Moxifloxacin	ND	ND	ND	0.5	0.5	0.25~1
Ciprofloxacin	ND	ND	ND	1	1	0.125~4
Gentamicin	ND	ND	ND	0.25	0.5	0.25~4
Rifampin	ND	ND	ND	0.125	16	0.125~64

MIC, minimum inhibitory concentration; S, susceptible; NS, nonsusceptible; MIC50, minimum inhibitory concentration at which 50% of isolates were inhibited; MIC90, minimum inhibitory concentration at which 90% of isolates were inhibited; MIC range, range of minimum inhibitory concentration. ND, Not determined.

## Discussion

Listeriosis is a rare foodborne illness in the elderly, immunosuppressive individuals, pregnant women and neonates, resulting in different clinical manifestations such as neurolisteriosis, bacteremia, and maternal-neonatal infections (Mylonakis et al., 2002; Dalton et al., 2011; Lamont et al., 2011; Mook et al., 2011; Goulet et al., 2012; De Noordhout et al., 2014; Wang et al., 2015; Charlier et al., 2017; Fan et al., 2019). *L. monocytogenes* belongs to facultative intracellular bacteria. The pregnancy diminishes a woman’s immune system and this is a predisposing factor for maternal and neonatal listeriosis. The microorganism can endanger the fetus *via* the placenta, resulting in preterm delivery, miscarriage, or stillbirth (Hong and Yang, 2012). Rarely, respiratory tract and cutaneous listeriosis were also documented (Morgand et al., 2018; Pilmis et al., 2020). The average prevalence of Chinese food products from 28 provinces was 4.42% from January 2008 to December 2016 (Li et al., 2018). However, comprehensive data concerning human listeriosis surveillance is lacking in China. Herein, we initiated a retrospective multi-center study, to better clarify the epidemiological characteristics of the pathogen and the clinical features of listeriosis in China.

The incidence of listeriosis varied geographically, 0.76/100.000 persons/year in Spain (Fernández Guerrero et al., 2012); 3.4/100,000 live births in the UK (Sapuan et al., 2017). The variable nature of *L. monocytogenes* hints that some listeriosis and deaths might go undiagnosed and unreported (Anand et al., 2016; Kylat et al., 2016; Fan et al., 2019). The perinatal incidence could also be higher due to undiagnosed spontaneous abortions and stillbirths (Kylat et al., 2016). *L. monocytogenes* meningitis yields a positive Gram stain in only about 33% of cases for the bacteria might decolorize readily during the Gram-staining procedure (Kylat et al., 2016). Also, the yield of the culture of CSF and blood decreased after treated with antibiotics in those with unconfirmed listeriosis (Anand et al., 2016).

*L. monocytogenes* may invade the bloodstream and central nervous system (Charlier et al., 2017; Fan et al., 2019; Chen et al., 2020). In our study, 75.0% and 14.6% were positive in blood and CSF culture. Comparatively, in 253 invasive listeriosis cases from 2011 to 2016 in China, bacteriemia and CNS infection accounted for 62.2% and 36.7%, respectively (Li et al., 2018). Another study in China from 1964 to 2010 also revealed that listeriosis presented predominantly as bacteriemia (68/147, 46%) and CNS infection (41/147, 31%) (Feng et al., 2013).

In nonmaternal-neonatal patients, listeriosis in other sites rarely occurred. In the present study, only 2 cutaneous listeriosis cases were observed, including a 63-year male with diabetes and a 22-year female, which accounted for 4.2% (2/48) of nonmaternal-neonatal listeriosis. Comparatively, according to a systemic review in mainland China, only one *L. monocytogenes* isolate was detected from wound exudate in 164 non-perinatal patients (1/164, 0.61%) (Fan et al., 2019). Similarly, between 1994 and 2018 in France, only 16 (0.2%) patients with skin/soft tissue listeriosis were identified. Of them, 11 (11/16, 69%) were male, and the median age was 62 (range, 8-93) years and 5(31%) patients reported immunosuppressive comorbidity (Pilmis et al., 2020).

Pregnant women are at high risk of listeriosis, approximately 12–20 times more prevalent than in the general population, resulting in a maternal, fetal, or live-born neonatal infection (Mylonakis et al., 2002; Elinav et al., 2014; Girard et al., 2014). Furthermore, there is a higher proportion of pregnancy-associated listeriosis in China than that reported in other countries (Goulet et al., 2012; Jensen et al., 2016). Maternal-neonatal listeriosis cases presented a serious threat. Of 26,221 deliveries, there were 5 cases of neonatal listeriosis (Wang et al., 2013). In our study, 66.7% were pregnancy-associated, 59.6% (31/52) experienced miscarriage or abortion, 9.1% (4/44) newborns were dead, but no pregnant women had central nervous system involvement or resulted in death, in agreement with the previous report (Wang et al., 2013). In a systematic review in China during the period of 2011–2017, perinatal listeriosis accounted for 59.6% (335/562), of which 32.68% resulted in abortion and/or newborn death (Fan et al., 2019). Another review through literature retrieval in China from 1964 to 2010 revealed 52% were pregnancy-related (Feng et al., 2013). Similarly, in a worldwide study from multiple centers, 5% (5/107) of pregnant women had an uneventful outcome, and 24% (26/107) of mothers experienced fetal loss (Charlier et al., 2017). Strategies should be in place to prevent pregnancy-associated listeriosis in those with predisposing factors (Bennion et al., 2008).

For the nonmaternal-neonatal infections, the incidence of listeriosis increases in the elderly population (Mook et al., 2011; Goulet et al., 2012; Birnie et al., 2019). The sex bias is rarely observed in nonmaternal listeriosis. In the current study, the median age of 48 nonmaternal-neonatal cases was 51.7 years, and 41.7% were female. Wang et al. reported that in a general hospital in China, the median age of the 25 adult nonmaternal listeriosis patients was 47 (range, 18–79) years with a female predominance (72%) (Wang et al., 2013). However, in Spain, the mean age of 64 listeriosis patients was 58.8 (range, 19 to 86) years, and 33% were female (Fernández Guerrero et al., 2012). This hinted that the age of listeriosis cases in China is younger than that in western countries, as explained partially by the different dietary habits (Charlier et al., 2017; Fan et al., 2019).

Some nonmaternal listeriosis patients might have risk factors (Mook et al., 2011). In our study, 64.6% listeria infections occurred in those with risk factors, compared with 74% (47 out of 64 sporadic listeriosis patients) in Spain (Fernández Guerrero et al., 2012), and 92% (23/25) in another study in China (Wang et al., 2013). These predisposing conditions often cover hematological and solid-organ neoplasia (leukemia, multiple myeloma, cancer, and metastases), autoimmune diseases (mainly SLE), cirrhosis of the liver, diabetes mellitus, chronic renal failure, the elderly and corticosteroids user (Fernández Guerrero et al., 2012; Wang et al., 2013; Friesema et al., 2015), in agreement with those observed in our study.

Listeriosis is documented to be associated with the highest case-fatality rate without early diagnosis and proper treatment, and its clinical presentation is often not specific (Birnie et al., 2019). In our study, the mortality rate was 10.4%. A previous systematic review, through literature retrieval in 28 provinces in China from 1964 to 2010, revealed that the overall listeriosis case-fatality rate was 26% (34/130) and 46% (21/46) were newborns (Feng et al., 2013). In another systematic review in China during the years 2011–2017, a mortality rate of 23.78% was detected in 227 non-perinatal listeriosis patients (Fan et al., 2019). In 253 invasive listeriosis cases were reported from 2011 to 2016 in 19 provinces, the overall case-fatality rate was 25.7% (stillbirth and abortion defined as deaths), with 26.4% in maternofetal cases and 24.4% in nonmaternofetal cases, respectively, while no deaths of pregnant women and children were reported (Li et al., 2018). In Israel during the year 2008–2014, the overall 30-day mortality of nonpregnancy-associated listeriosis patients was 20.5% (39/190) (Dickstein et al., 2019). In Denmark during 2002–2012, the overall mortality for non-pregnancy-associated cases was 27%, and the mortality rate varied with age: 22% for patients <70 years of age versus 33% for patients >70 years (Jensen et al., 2016). The lower case-fatality rate in our study might be explained by the fact that our subjects were younger and the bias due to the small size of the study. The conclusions should be interpreted with caution.

Three serogroups of *L. monocytogenes* isolates, namely IIa, IIb, and IVb, caused the majority of clinical cases (Li et al., 2018). In our study, four serogroups were identified, and IIb predominated (76, 52.7% strains), followed by IIa (42, 29.2%), IVb (25, 17.4%) and IIc (1, 0.7%). Similarly, in a small-sized study in China, in the 28 clinical listeria isolates, the most prevalent serotype was 1/2b with a frequency of 64.3%, followed by 1/2a (21.4%), 4b (7.1%), 1/2c (3.6%), and 3a (3.6%) (Wang et al., 2015). In a systemic review of listeriosis in mainland China, in 87 clinical Listeria isolates with serogroup data available, IIb (serovars 1/2b, 38/87, 43.68%) and IIa (serovars 1/2a, 24/87, 27.59%) were prevalent (Fan et al., 2019).

MLST is often applied in the evaluation of the clonal relationship among *L. monocytogenes* strains, which are geographically varied and change over time (Lee et al., 2012; Elinav et al., 2014; Henri et al., 2016; Jensen et al., 2016; Maury et al., 2016). The distribution of STs in our study was similar with the previous studies: for the clinical *L. monocytogenes* isolates collected from patients in China, ST87 was the most prevalent ST type (Wang et al., 2015; Fan et al., 2019; Zhang et al., 2019; Yin et al., 2020). However, the clinical *L. monocytogenes* isolates circulating in China constructed distinct clusters that distributed across the phylogenetic tree of the worldwide isolates: ST87 is seldom linked to human infection cases in other countries (Althaus et al., 2014; Yin et al., 2020). In Austria, the three most frequent STs were ST1 (7/31; 22.6%), ST155 (4/31; 12.9%), and ST451 (3/31; 9.7%) (Cabal et al., 2019). In Switzerland from 2011 to 2013, 93 isolates were distinguished into 35 STs, and ST8 (14, 15.1%) and ST1 (14, 15.1%) were the most prevalent (Althaus et al., 2014). However, when it comes to food isolates, in 80 *L. monocytogenes* isolates obtained from retail ready-to-eat food in China, ST8 (24/80,30%) and ST1(16/80, 20%) were isolated most frequently, and only seven ST87 (8.75%) isolates were detected (Wu et al., 2016). This was also confirmed in another study in China, in which ST87, ST3, and ST7 were the main STs in human listeriosis strains, and ST9 and ST8 predominated in food isolates (Li et al., 2018). The high frequency of ST87 constitutes the notable feature of clinical *L. monocytogenes* strains in China, this might be partially explained by multiple virulence determinants harbored in many ST87 isolates (Yin et al., 2020). Specific CCs have been associated with severe listeriosis cases. As previously reported, CC1 and CC4 were associated with maternal-neonatal listeriosis and neurolisteriosis with few or no immunosuppressive comorbidities; the food-associated clones CC9 and CC121 were more often isolated in highly immunocompromised patients (Maury et al., 2016). However, the *L. monocytogenes* isolates in Maury et al.’s study were collected only in France between January 2005 and October 2013, and its conclusion is difficult to extrapolate to other countries. In line with our data, we did not detect the correlation between ST/CC and clinical listeriosis presentation, in agreement with a report in Switzerland (Althaus et al., 2014).

To date, *L. monocytogenes* isolates represent four phylogenetic lineages, mainly I and II, that can be subdivided into multiple CCs and STs and are groups of genetically-related *L. monocytogenes* strains originating from a recent common ancestor (Lee et al., 2012; Nyarko and Donnelly, 2015). In our study, *L. monocytogenes* strains showed a high degree of genetic diversity: 23 STs were distinguished, and 101 (70.1%) and 43 (29.9%) strains belong to lineage I and II respectively. In accordance with the previous studies, the clinical invasive listeriosis cases mainly belonged to lineage I strains, while most of the food and environmental strains were attributed to lineage II, however, recent studies have revealed that the hypervirulent strains associated with invasive listeriosis were also from lineage II (Charlier et al., 2017; Cardenas-Alvarez et al., 2019). Similarly, the *L. monocytogenes* strains in our study were almost collected from those with invasive listeriosis, and the strains belonging to lineage II (e.g., CC7 and CC14) were identified indeed.

Mortality of listeriosis mainly depended on the severity of the underlying disease and timely, efficient treatment. The most effective regimen is currently based on a synergistic association of high doses of ampicillin (or amoxicillin) and gentamicin due to a synergistic effect (Hof, 2004). Although rifampin, vancomycin, linezolid, and carbapenems have been proposed as a possible alternative therapy for listeriosis. Our AST results demonstrate that all *L. monocytogenes* strains were uniformly susceptible to penicillin, ampicillin, and meropenem, and a low MIC for vancomycin and moxifloxacin was also noted, similar to the data in France, Spain, and Switzerland (Morvan et al., 2010; Fernández Guerrero et al., 2012; Althaus et al., 2014). In line with our results, aminopenicillin should be considered as the drug of choice and recommended in mainland China, and AST is not required. Gentamicin is contraindicated in pregnant women, and its MICs were 8µg/ml in 4 *L. monocytogenes* isolates in our study, which should be used with caution if combined with aminopenicillin in specific cases. TMP-SMX is generally used in case of allergy to beta-lactams (Morvan et al., 2010; Fernández Guerrero et al., 2012), but the experimental data are far from conclusive (Hof, 2004). In our study, one *L. monocytogenes* isolate was nonsusceptible to TMP-SMX with a MIC value of 4/762 µg/ml. Rifampin might penetrate well into the host and be used with other antibiotics in recommendations (Hof, 2004; Fernández Guerrero et al., 2012). In our study, we observed the MICs of rifampin were elevated in 26 *L. monocytogenes* strains (one with a MIC value of 2µg/ml, 3 of 8µg/ml, and 16 of 16µg/ml). Furthermore, Viviane Chenal-Francisque et al. previously reported an *L. monocytogenes* clinical isolate was highly resistant to rifampin, with its MIC >32 µg/ml, mediated mostly by mutations in *rpoB* (Chenal-Francisque et al., 2014). This demonstrated that the MIC of rifampin should be determined before use or not used alone.

In summary, the data from our study represented the diversity of the entire collection and the genetically distinct lineages of many *L. monocytogenes* strains recovered from multiple sources in mainland China, which might be underrecognized previously. A relatively low onset age in nonmaternal-neonatal listeriosis and a high prevalence of ST87 were notable features of our clinical *L. monocytogenes* strains. The study has provided useful evidence for the implementation of effective listeriosis treatment and prevention strategies.

## Data Availability Statement

The original contributions presented in the study are included in the article/**Supplementary Material**. Further inquiries can be directed to the corresponding author.

## Author Contributions

BL, JY, CG, DL, YC, LH, XC, AW, YLL, YL, ZZ, MJ, HX, YS, BF, LX, QY, YN, LW, CB GL, HW,TY, CL, MT, JW, WG, JZ, and WZ isolated the L. monocytogenes isolates, performed the tests, and collected the clinical and laboratory data. BL, DW, and DL made substantial contributions to conception and design, and drafted the manuscript. All authors contributed to the article and approved the submitted version.

## Funding

This study was supported by Beijing Municipal Science & Technology Commission, PR China (No. Z171100001017118) for specimen’s collection and analysis.

## Conflict of Interest

The authors declare that the research was conducted in the absence of any commercial or financial relationships that could be construed as a potential conflict of interest.

## Supplementary Material

Supplementary Figure 1 Geographical locations and distribution of *L. monocytogenes* strains from patients diagnosed with listeriosis in 15 cities/provinces in China between 2008 and 2019. The color-highlighted cities/provinces represent those where *L. monocytogenes* were isolated, with the number of strains shown in brackets.
