# Supplementary figures and images for: Listeriosis Cases and Genetic Diversity of Their L. monocytogenes Isolates in China, 2008–2019
Source: Front Cell Infect Microbiol. 2021 Feb 19;11:608352. doi: 10.3389/fcimb.2021.608352 (PMC7933659; doi:10.3389/fcimb.2021.608352)

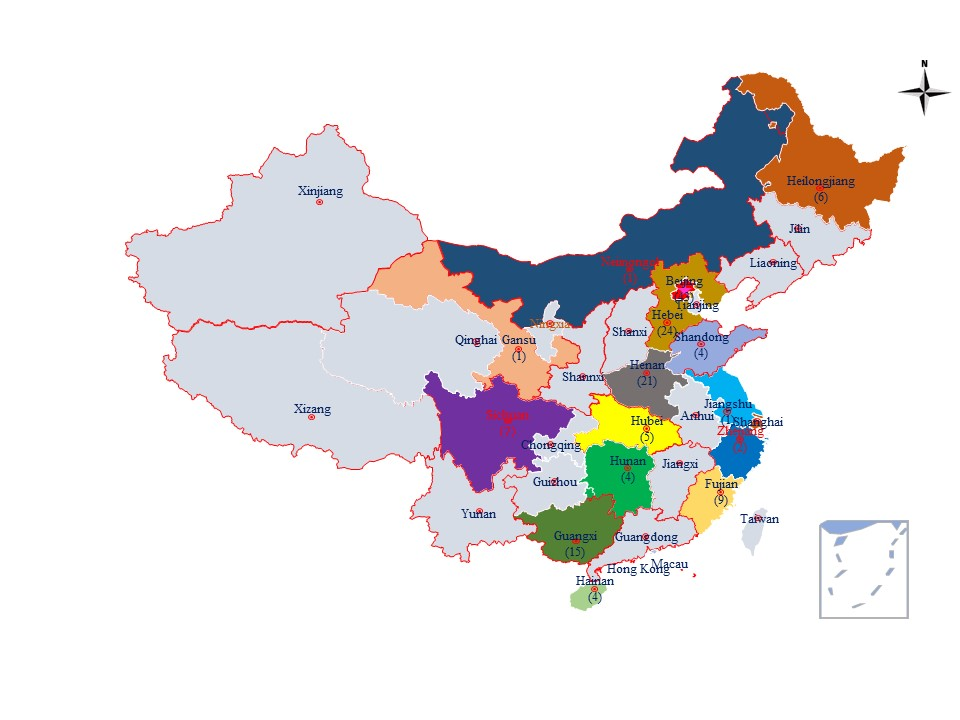

Supplement: Supplementary Figure 1 — Geographical locations and distribution of L. monocytogenes strains from patients diagnosed with listeriosis in 15 cities/provinces in China between 2008 and 2019. The color-highlighted cities/provinces represent those where L. monocytogenes were isolated, with the number of strains shown in brackets. [file Image_1.tif]
